# Supplementary material for: The Cluster Structure Function
Source: IEEE Trans Pattern Anal Mach Intell. Author manuscript; Available in PMC 2023 Sep 27. (PMC10525042; doi:10.1109/TPAMI.2023.3264690)
Supplement: supp1-3264690 [file NIHMS1923207-supplement-supp1-3264690.pdf]

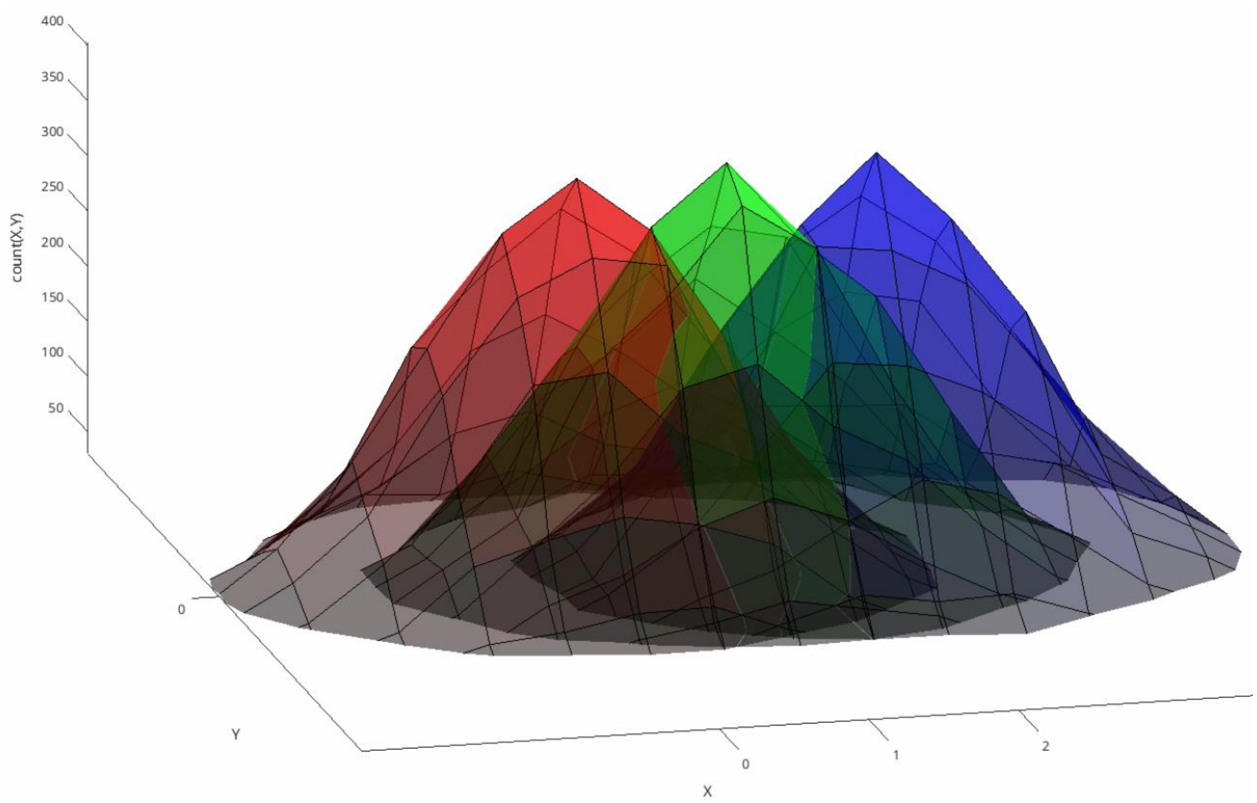

Supplementary Figure 1: Histogram for synthetic dataset containing 3 clusters. Here cluster spacing equals 1.0. Each cluster is shown in red, green, blue. The data was generated from a mixture of 3 normal distribution with means $\mu = [0, 1, 2]$ and identical covariance $\Sigma = [1, 0; 0, 1]$.
